# Mindful Parenting and Emotional and Behavioural Problems in Children with Autism Spectrum Disorder: The Chain Mediating Role of Parenting Stress and Family Management

**DOI:** 10.3390/bs16091614

**Published:** 2026-09-10

**Authors:** Ting Huang, Jingyi Wang, Yilin Li, Ou Chen

**Affiliations:** 1 School of Nursing and Rehabilitation, Shandong University, Jinan 250012, China; 202536923@mail.sdu.edu.cn (T.H.); 202136688@mail.sdu.edu.cn (J.W.); 202536928@mail.sdu.edu.cn (Y.L.); 2 Qingdao Mental Health Center, Qingdao 266034, China

**Keywords:** emotional and behavioural problems, autism spectrum disorder, children, mindful parenting, parenting stress, family management

## Abstract

Autism spectrum disorder (ASD) has a continuously rising global prevalence, imposing a heavy burden on affected families and society. Emotional and behavioural problems are the most common comorbidities of ASD and severely impair the long-term prognosis of affected children. While mindful parenting represents a validated positive parenting model, the internal mechanisms underlying its association with emotional and behavioural developmental outcomes in children with ASD remain unclear. This study adopted a cross-sectional design. Between April and August 2023, 268 parent–child dyads were recruited via convenience sampling from hospitals and special education settings in Jinan, China. Validated instruments were used to assess mindful parenting, parenting stress, family management, and child emotional and behavioural problems, and data were analysed using path analysis with observed variables. The results showed that the sequential chain mediation pathway was fully consistent with the data for total difficulties, with the total indirect effect accounting for 66.71% of the total association. For prosocial behaviour, the model received only partial support, with a 61.54% total indirect association, as the unique indirect path via parenting stress did not reach statistical significance. These findings indicate that mindful parenting is associated with fewer child difficulties through a sequence involving reduced parenting stress and better family management. For prosocial behaviour, this association appears primarily linked to family management, with no significant unique contribution of parenting stress. The study reveals potential family process mechanisms and can inform the development of family-centred interventions.

## 1. Introduction

Autism spectrum disorder (ASD) is a neurodevelopmental disorder characterised by impaired social interaction and communication, restricted interests, and repetitive stereotypic behaviours (Lord et al., 2020). In recent years, the number of children diagnosed with ASD worldwide has continued to rise (Hirota & King, 2023). Epidemiological surveys indicate that the global prevalence is approximately 1%, with a higher rate in China of about 1.8% (Zeidan et al., 2022; Zhao et al., 2023). The disorder typically manifests before the age of three years, is frequently accompanied by multiple comorbidities, and currently has no curative treatment (Hirota & King, 2023; Pascoe et al., 2023); its physical and psychological impact can persist throughout life, necessitating long-term care and support and imposing a heavy burden on families (Dückert et al., 2023). Studies indicate that the economic burden of ASD in China is substantial, with per capita lifetime costs of up to $2.65 million and an annual national economic burden of $41.8 billion in 2020, exerting immense pressure on families and society (Zhao et al., 2024). Thus, ASD has become a serious public health challenge.

Emotional and behavioural problems are the most common comorbid symptoms of ASD and can be divided into internalising and externalising problems. Internalising problems mainly manifest as anxiety, low mood, withdrawal, and irritability, while externalising problems present as hyperactivity, inattention, impulsivity, and aggression (Li et al., 2020). These problems, which persist over the course of the disorder, not only severely impede social development, rehabilitation outcomes, and social adaptation, but also affect long-term prognosis and quality of life (Hossain et al., 2020). A study of Chinese children with ASD found that emotional and behavioural problems can lead to isolation, disruption of education and therapeutic interventions, and restricted participation in social and community activities, thereby exacerbating functional impairment (Tan et al., 2022). Therefore, attention to emotional and behavioural problems in children with ASD is crucial for improving disorder management.

The family is the primary setting for the growth and rehabilitation of children with ASD, and parents, as the main caregivers, adopt parenting styles that constitute an important environmental factor influencing the emotional and behavioural development of these children (Marshall et al., 2025). Existing research indicates that positive parenting styles are associated with a lower risk of emotional and behavioural problems and better long-term developmental outcomes (Tarver et al., 2019; Kulasinghe et al., 2023). Moreover, the Healthy China Action Plan (2019–2030) explicitly calls for strengthening early intervention for child psychological and behavioural problems, improving the family support and care service system for children with special needs, and enhancing family caregiving capacity (The State Council of the People’s Republic of China, 2019). Therefore, within this national context, exploring effective positive parenting models holds practical significance for ameliorating emotional and behavioural problems in these children.

Mindful parenting is a positive parenting model that integrates mindfulness principles into the rearing process, emphasising that caregivers consciously, nonjudgmentally, and with full acceptance observe their own and their children’s emotions and needs during moment-to-moment interactions (Duncan et al., 2009). In recent years, this model has become a research focus in the field of family intervention for children with special needs (Ahemaitijiang et al., 2021). A study by Chan et al. (2022) in China noted that higher levels of mindful parenting are associated with better parental responsiveness to children’s needs, fewer negative parenting behaviours, and a more stable and supportive family environment, which corresponds to fewer emotional and behavioural problems. Hence, this more accepting and positive parenting approach may be associated with fewer emotional and behavioural problems in children with ASD.

However, most existing research on mindful parenting, both domestically and internationally, has focused on its direct effects on core symptoms of ASD and parental mental health. At the same time, explorations of its relationship with emotional and behavioural problems in children with ASD remain scarce (Peng et al., 2025). On the one hand, although many studies have examined this relationship, they have predominantly focused on typically developing children or adolescents, with little attention to the specific population of children with ASD (Yang et al., 2022; Lam et al., 2025); on the other hand, owing to variations in study designs, existing research has not reached a consensus on this relationship (Neece et al., 2024; McKinney et al., 2025). Therefore, it is necessary to further clarify the relationship between mindful parenting and emotional and behavioural problems in children with ASD and its underlying mechanisms within ASD families, which holds significant theoretical and practical implications for optimising family intervention programmes, enhancing family support systems, and promoting the physical and mental development of these children.

Parenting stress refers to the negative psychological experience arising from caregiving responsibilities, parenting challenges, and role expectations during the parenting process, a prevalent psychological issue among parents of children with ASD (Barroso et al., 2018). Existing research has confirmed that parenting stress is significantly positively correlated with emotional and behavioural problems in children with ASD (Rodriguez et al., 2019). When parents experience chronically high parenting stress, they are prone to emotional dysregulation and negative parenting behaviours, which impair the quality of parent–child interaction and, in turn, exacerbate children’s anxiety and problem behaviours (Suvarna et al., 2024). In contrast, mindful parenting can effectively alleviate parenting stress and improve parenting behaviours (Mo et al., 2024; Peng et al., 2025).

Family management refers to a series of systematic caregiving strategies and management behaviours adopted by families to address the long-term rehabilitation needs of the child and promote development, serving as a key family factor influencing rehabilitation outcomes and developmental trajectories in children with ASD (Fitzgerald et al., 2025). The extant literature indicates that family management plays an important role between parental parenting states and child developmental outcomes; that is, positive parenting states can effectively enhance family management, establish a stable rehabilitation environment and daily routine for the child, and thereby alleviate emotional and behavioural problems (John & Hemalatha, 2026). Family systems theory posits that parental psychological states directly affect family functioning and the implementation of management behaviours, which in turn influence children’s growth and development (Piro-Gambetti et al., 2024, 2025).

In summary, previous research has confirmed that mindful parenting, parenting stress, and family management all influence emotional and behavioural problems in children with ASD, yet the specific pathways and internal mechanisms through which these three factors jointly operate remain unclear. Furthermore, studies from different countries exhibit considerable heterogeneity, and ASD has its own particularities, whilst parents of children with ASD face far greater parenting challenges than parents of typically developing children (Fan & Ko, 2025). Within the Chinese cultural context, specifically exploring the association between mindful parenting and emotional and behavioural problems in children with ASD, and conducting an in-depth analysis from a family perspective, therefore holds important practical value for the rehabilitation of these children. The rehabilitation of children with ASD relies on multidisciplinary teamwork, and mindful parenting, parenting stress, and family management are areas in which rehabilitation professionals, including nurses, psychologists, and therapists, can directly intervene. Nevertheless, empirical evidence on this pathway remains very limited within rehabilitation contexts, and it cannot yet provide specific guidance for clinical practice. Therefore, this study aims to construct a chain mediation model to reveal the internal mechanism by which mindful parenting affects emotional and behavioural problems in children with ASD, and to provide empirical evidence for rehabilitation professionals to implement family-centred care, thereby filling this gap in practice.

### 1.1. Theoretical Background

This study is underpinned by the Individual and Family Self-Management Theory (IFSMT) to elucidate the mechanism by which mindful parenting affects emotional and behavioural problems in children with ASD. Proposed by Ryan and Sawin (2009), this theory aims to purposefully incorporate health-related behaviours into the daily routines of individuals or families. IFSMT defines self-management as a complex, dynamic phenomenon encompassing context, process, and outcome dimensions (Bonis & Sawin, 2016). Specifically, the context dimension refers to risk and protective factors in the self-management process, including condition-specific factors, physical or social environments, and individual and family characteristics; the process dimension focuses on the mechanisms of self-management behaviours, involving knowledge and beliefs, self-regulation, and social facilitation; and the outcome dimension is divided into proximal outcomes (such as self-management behaviours and healthcare costs) and distal outcomes (such as health status, quality of life, and medical expenses). IFSMT clearly delineates the relationships among these dimensions: context factors can influence the self-management process (i.e., the process dimension) as well as directly affect proximal or distal outcomes, whereas process factors also affect proximal or distal outcomes. This theory can not only guide the design of intervention programmes and measurement instruments but also support testing for mediating and moderating effects among the proposed relationships, and has thus been widely applied in various research designs (Bauer et al., 2025).

**Figure 1:**
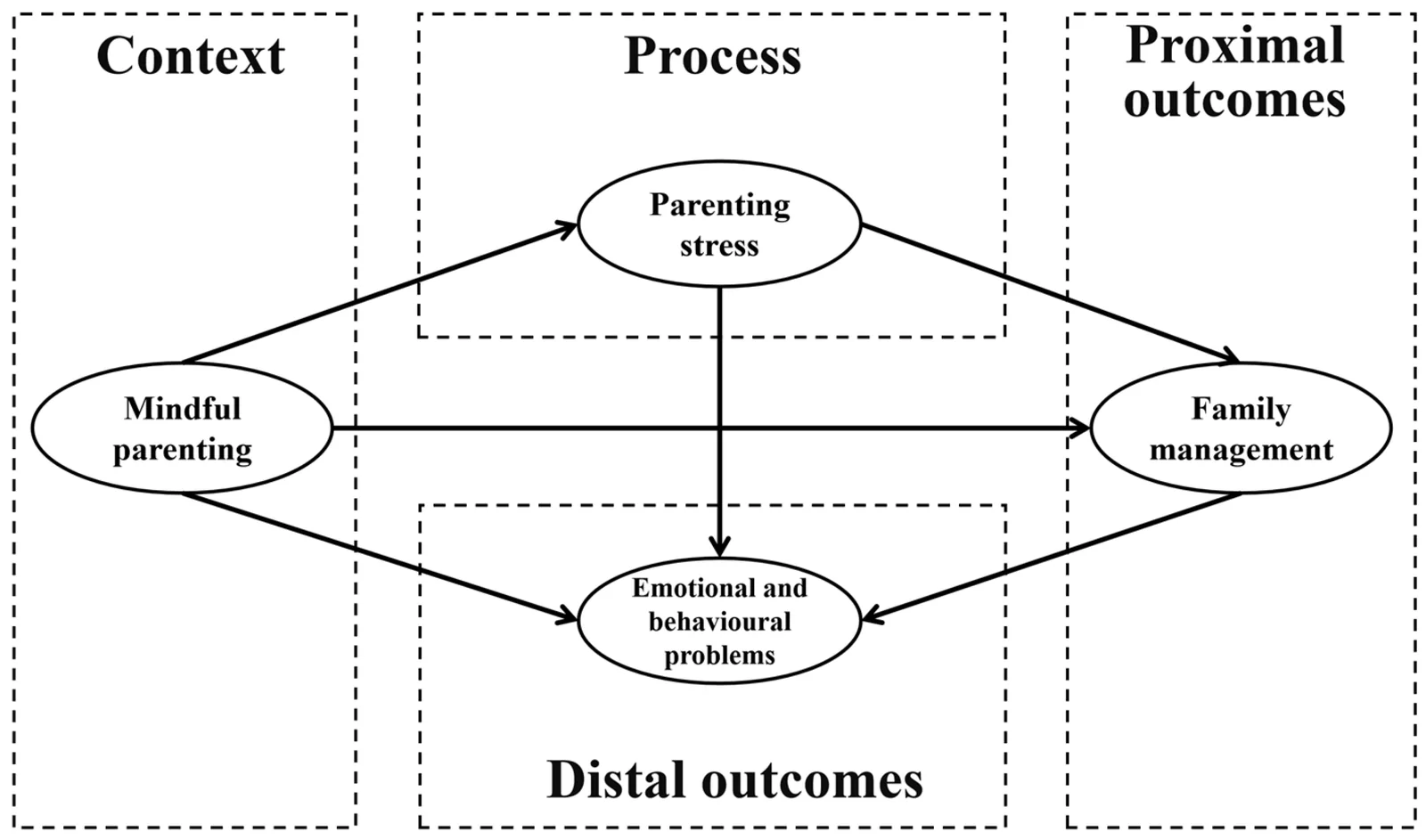
The hypothetical theoretical model.

Based on this theory, within the context of long-term care required by children with ASD, mindful parenting, as a positive and objective parenting style, aligns with the individual and family factors in the context dimension; parenting stress, involving the management of emotional and cognitive responses related to health behaviour change, corresponds to the self-regulation component of the process dimension; family management, defined as the adjustment behaviours through which the family integrates the child’s disorder management into daily life, constitutes an important element of self-management behaviours in proximal outcomes; and the child’s emotional and behavioural problems, the most common comorbid symptoms of ASD, represent health status in distal outcomes. Accordingly, this study sets mindful parenting as the independent variable, the emotional and behavioural problems of children with ASD as the dependent variable, and parenting stress and family management as chain mediating variables, thereby constructing a chain mediation theoretical model (see Figure 1).

### 1.2. Research Hypothesis

**Hypothesis 1.**
  *Mindful parenting is negatively correlated with emotional and behavioural problems in children with ASD.*

In this study, mindful parenting refers to a parenting model in which parents integrate mindful awareness and nonjudgmental acceptance into the overall upbringing of children with ASD, flexibly address parenting challenges, and actively respond to the child’s needs. Extant research indicates that mindful parenting is associated with fewer impulsive and negative parental reactions to children’s problem behaviours, higher quality of parent–child interactions, and a more supportive family environment, which may correspond to fewer emotional and behavioural problems (Aydin, 2023). It is thus hypothesised that the higher the level of parental mindful parenting, the fewer emotional and behavioural problems in children with ASD.

**Hypothesis 2.**
  *Parenting stress mediates the relationship between mindful parenting and emotional and behavioural problems in children with ASD.*

In this study, parenting stress specifically refers to the psychological stress and negative emotional experiences perceived by parents of children with ASD during long-term caregiving as a result of parenting difficulties and caregiving burdens. Parenting stress is closely associated with emotional and behavioural problems in children; the higher the perceived parenting stress, the more likely parents are to adopt negative parenting behaviours, leading to more severe emotional and behavioural problems in children (Yorke et al., 2018). A systematic review found that mindful parenting can significantly reduce parenting stress among parents of children with special needs, acting as an important antecedent of parenting stress (Mo et al., 2024). Based on this, we hypothesise that parenting stress mediates the association between mindful parenting and emotional and behavioural problems in children with ASD; higher mindful parenting is associated with lower parenting stress, which in turn corresponds to fewer child emotional and behavioural problems.

**Hypothesis 3.**
  *Family management mediates the relationship between mindful parenting and emotional and behavioural problems in children with ASD.*

In this study, family management specifically refers to systematic management behaviours undertaken by ASD families to address the child’s rehabilitation needs and promote development, including care plan formulation, rehabilitation intervention implementation, and family functioning adjustment. Research indicates that parenting style is associated with family management behaviours; higher levels of mindful parenting correspond to better family management capabilities, which may be related to a more stable and orderly caregiving environment for the child (Chan et al., 2022). Meanwhile, multiple studies consistently show that effective family management can significantly reduce emotional and behavioural problems in children with ASD, providing strong support for rehabilitation and development (Fitzgerald et al., 2025; Ibrahim et al., 2025). It is thus speculated that family management mediates the association between mindful parenting and emotional and behavioural problems in children with ASD.

**Hypothesis 4.**
  *Parenting stress and family management mediate the relationship between mindful parenting and emotional and behavioural problems in children with ASD.*

Existing research confirms a significant negative association between parenting stress and family management (Fitzgerald et al., 2025) and that family management may mediate the link between parenting stress and developmental outcomes in children with special needs (Hou et al., 2023). This logic aligns closely with the Individual and Family Self-Management Theory: mindful parenting is associated with lower parenting stress; when parents experience less psychological burden, they may have more resources for family caregiving management, which corresponds to better family management quality and, in turn, a more supportive growth environment and fewer emotional and behavioural problems in children with ASD. Combined with the preceding hypotheses, it can be inferred that parenting stress and family management play a chain mediating role in the association between mindful parenting and emotional and behavioural problems in children with ASD.

## 2. Method

### 2.1. Study Design

This was a cross-sectional study utilising the convenience sampling method. The reporting of findings strictly adhered to the Strengthening the Reporting of Observational Studies in Epidemiology (STROBE) guidelines to ensure methodological rigour and transparency (von Elm et al., 2007).

### 2.2. Participants

This investigation was conducted between April and August 2023 in the departments of child healthcare of 3 tertiary Grade A comprehensive hospitals and 3 special education institutions in Jinan, China. Children with ASD and their parents were recruited as study participants, with only one parent (father or mother) enrolled for each eligible child to ensure data independence and representativeness.

The inclusion criteria for the children were as follows: (1) aged 3 to 14 years; (2) met the diagnostic criteria for ASD according to the Diagnostic and Statistical Manual of Mental Disorders, 5th edn. (DSM-5); (3) had a disorder duration of no less than 6 months. Exclusion criteria for the children: (1) complicated with other comorbid mental disorders; (2) complicated with life-threatening severe diseases.

The inclusion criteria for the parents were as follows: (1) aged 22 years and over; (2) being the biological father or mother of the enrolled child with ASD; (3) had been the primary caregiver of the child since the ASD diagnosis; (4) possessed basic literacy skills and the ability to complete the questionnaire independently; (5) provided informed consent and voluntarily participated in this study. Exclusion criteria for the parents: (1) concurrently participating in other similar studies; (2) with severe chronic diseases (e.g., cerebrovascular disease, autoimmune disease, malignant tumours) or diagnosed mental disorders; (3) withdrew voluntarily during the survey process.

### 2.3. Sample Size

The sample size was estimated according to Kendall’s sample size estimation principle for questionnaire-based studies, which requires the sample size to be 5 to 10 times the number of questionnaire items (Preacher & Kelley, 2011). In this study, a total of 29 items were included in the questionnaires; the minimum required sample size was 145. In addition, the sample size was also estimated using an online a priori sample size calculator. Parameters included a power level of 0.95, an anticipated effect size of 0.3, a significance level of 0.05, 4 latent variables, and 29 observed variables, resulting in a minimum required sample size of 188 participants. Furthermore, path analysis requires more than 200 samples (Lei & Wu, 2007). Considering the possibility of invalid questionnaires, the sample size was increased by 20%, and the final target sample size of this study was set at 240 cases. Ultimately, 268 valid responses were obtained. A post hoc power analysis confirmed that the final sample size of 268 met the power requirements for path analysis, ensuring robust statistical evaluation of the relationships among mindful parenting, parenting stress, family management, and emotional and behavioural problems in children with autism spectrum disorder.

### 2.4. Measurements

#### 2.4.1. Demographic Data Questionnaire

Demographic characteristics of the participants included child characteristics and parental characteristics. Child characteristics comprised age, gender, disorder duration, severity of the condition, and whether the child was an only child. Parental characteristics comprised relationship to the child (father or mother), age, gender, place of residence, educational level, employment status, monthly household income per capita, monthly expenditure on the child’s rehabilitation, and duration of continuous care for the child.

#### 2.4.2. Parenting and Emotional and Behavioural Problems

The Strengths and Difficulties Questionnaire (SDQ), developed by Goodman (1997), was used to screen for emotional and behavioural problems in children and adolescents aged 3 to 16 years, and has demonstrated appropriate reliability and validity. This study adopted the Chinese version revised by Kou et al. (2005). The questionnaire comprises 25 items in five dimensions: emotional symptoms (5 items), conduct problems (5 items), hyperactivity/inattention (5 items), peer relationship problems (5 items), and prosocial behaviour (5 items). Each item is scored on a 3-point Likert scale ranging from 0 (not true) to 2 (certainly true); five items are reverse-scored. According to the cut-off criteria established by Kou et al. (2005), for emotional symptoms a score of 0–3 is classified as normal, 4 as borderline, and 5–10 as abnormal; for conduct problems, 0–2 is normal, 3 borderline, and 4–10 abnormal; for hyperactivity/inattention, 0–5 is normal, 6 borderline, and 7–10 abnormal; for peer relationship problems, 0–2 is normal, 3 borderline, and 4–10 abnormal; and for prosocial behaviour, 6–10 is normal, 5 borderline, and 0–4 abnormal. The total difficulties score is derived by summing the four problem subscales, yielding a range of 0 to 40, with higher scores indicating greater levels of difficulty. A total difficulties score of 17 to 40 is considered to indicate the presence of emotional and behavioural problems. The prosocial behaviour score ranges from 0 to 10, with higher scores reflecting better prosocial skills. The parent-report version was used in this study, which is suitable for assessing emotional and behavioural problems in children with ASD (Gu et al., 2023). Both the total difficulties score and the prosocial behaviour score were used to comprehensively reflect the child’s emotional and behavioural abnormalities (Xu et al., 2019). Confirmatory Factor Analysis (CFA) supported the original five-factor structure of the SDQ in the present sample, with acceptable model fit: chi-square to degrees of freedom ratio (*χ*^2^/*df*) = 2.241, Comparative Fit Index (CFI) = 0.942, Tucker–Lewis Index (TLI) = 0.931, Root Mean Square Error of Approximation (RMSEA) = 0.068, Standardized Root Mean Square Residual (SRMR) = 0.057. In this study, the Cronbach’s *α* for the total difficulties scale was 0.917, and that for the prosocial behaviour scale was 0.80.

#### 2.4.3. Mindful Parenting

The Mindfulness in Parenting Questionnaire (MIPQ), developed by McCaffrey et al. (2017), was used to assess the level of mindful parenting in parents of children aged 2 to 16 years, and has demonstrated appropriate reliability and validity. This study adopted the Chinese version revised by Wu et al. (2019). The questionnaire comprises 28 items in two dimensions: being present with mindfulness (13 items) and mindful discipline (15 items). Each item is scored on a 4-point Likert scale ranging from 1 (occasionally) to 4 (always). Total scores range from 28 to 112, with higher scores indicating higher levels of mindful parenting. CFA supported the original two-factor structure of the MIPQ in the present sample, with good model fit: *χ*^2^/*df* = 2.087, CFI = 0.951, TLI = 0.945, RMSEA = 0.064, SRMR = 0.052. In this study, the Cronbach’s *α* for the scale was 0.967.

#### 2.4.4. Parenting Stress

The Parenting Stress Index-Short Form (PSI-SF-15), developed by Abidin (1990), was used to assess parenting stress in parents and has demonstrated appropriate reliability and validity. This study adopted the Chinese version revised by Luo et al. (2021). The scale comprises 15 items in three dimensions: parental distress, parent–child dysfunctional interaction, and difficult child. Each item is scored on a 5-point Likert scale ranging from 1 (strongly disagree) to 5 (strongly agree). Total scores range from 15 to 75, with higher scores indicating higher levels of perceived parenting stress. CFA supported the original three-factor structure of the PSI-SF-15 in the present sample, with acceptable model fit: *χ*^2^/*df* = 2.415, CFI = 0.935, TLI = 0.918, RMSEA = 0.073, SRMR = 0.062. In this study, the Cronbach’s *α* for the scale was 0.930.

#### 2.4.5. Family Management

The Family Management Measure (FaMM), developed by K. A. Knafl et al. (2010), was used to assess family responses to managing a child’s chronic condition and has demonstrated appropriate reliability and validity. This study adopted the Chinese version introduced and revised by Zhang and Wei (2009). The measure comprises 53 items in six dimensions: child daily life (5 items), condition management ability (12 items), parental mutual support (8 items), condition-related worry (10 items), condition management difficulty (14 items), and condition management burden (4 items). Each item is scored on a 5-point Likert scale ranging from 1 (strongly disagree) to 5 (strongly agree), with some items reverse-scored. The total score ranges from 53 to 265. In this study, the six dimensions were categorised into two higher-order domains: positive family management assessment covering child daily life, condition management ability and parental mutual support, and negative family management assessment covering condition-related worry, condition management difficulty and condition management burden. This two-domain structure aligns with the original theoretical framework of the scale, which distinguishes between family management resources and condition-related burdens as two relatively independent conceptual domains rather than opposite ends of a single continuum (K. Knafl et al., 2011), and this classification approach has been widely adopted in prior empirical studies (Sutthisompohn & Kusol, 2021; Zhang & Wei, 2009). Higher scores on the positive domain indicate better family management capacity, while higher scores on the negative domain indicate greater management difficulty and burden. A second-order CFA was conducted to verify the structural validity of this two-factor higher-order structure in the present sample, and the results showed acceptable model fit: *χ*^2^/*df* = 2.683, CFI = 0.926, TLI = 0.917, RMSEA = 0.079, SRMR = 0.068. In this study, Cronbach’s *α* for the positive assessment subscale was 0.956, and for the negative assessment subscale it was 0.960.

### 2.5. Data Collection

Before the formal survey’s commencement, all researchers received standardised unified training, and official consent for the survey was obtained from the administrative department of the participating medical institutions and special education institutions. Before the survey, the purpose, significance, completion method, and precautions of the study were clearly introduced to the eligible participants, and the survey was conducted only after the participants provided written informed consent. During the survey, standardised unified instructions were used to explain the requirements for questionnaire completion, and the researchers answered participants’ questions in a standardised manner throughout the process. All questionnaires were collected and checked on the spot by the researchers, and any omissions, logical errors, or unclear answers were promptly verified and corrected with the participants.

A total of 275 questionnaires were distributed to eligible participants during the study period. After excluding invalid questionnaires (including incomplete questionnaires, questionnaires with identical answers to all items, and questionnaires completed in less than 5 min), 268 valid questionnaires were finally obtained, resulting in an effective response rate of 97.5%.

### 2.6. Statistical Analysis

This study employed IBM SPSS Statistics 27.0 and Mplus 8.3 for data analysis. A two-tailed *p* < 0.05 was considered to indicate a statistically significant difference. Initially, common method bias was tested using the Harman single-factor test (Podsakoff et al., 2003). Next, categorical data were summarised using frequencies and percentages, while continuous variables were tested for normality via skewness and kurtosis statistics. All continuous variables followed an approximate normal distribution and were therefore presented as mean ± standard deviation. Subsequently, independent samples *t*-tests and one-way analysis of variance were conducted to compare between-group differences in total difficulties score and total prosocial behaviour score of children with ASD across different sociodemographic characteristics. Following this, Pearson’s correlation analysis was performed to examine the bivariate associations between mindful parenting, parenting stress, and positive and negative family management assessment scores. Finally, a path analysis with observed variables was specified based on the IFSMT, with the Robust Maximum Likelihood (MLR) estimator applied to examine the mechanism underlying the association between mindful parenting and children’s emotional and behavioural problems. All constructs were treated as observed variables using scale total scores. To reduce potential confounding bias, demographic variables that showed significant associations with the outcome variables in the univariate analyses were included as covariates in the path model. All path coefficients and mediating effects reported in the manuscript are adjusted for these covariates. Good model fit was determined according to the following criteria: *χ*^2^/*df* < 3, CFI > 0.90, TLI > 0.90, RMSEA < 0.08, and SRMR < 0.08 (Hu & Bentler, 1999). To empirically justify the model specification, we compared two nested path models for each outcome variable. The restricted model included only the sequential chain pathway X → M1 → M2 → Y, with all direct paths from X and M1 to Y constrained to zero. The saturated partial mediation model released all direct paths for free estimation. Satorra–Bentler scaled chi-square difference tests were conducted to compare model fit, which is compatible with the MLR estimator. For the total difficulties model, the saturated model showed significantly better fit than the restricted model, supporting the inclusion of the direct path from parenting stress to child difficulties. For the prosocial behaviour model, no significant fit difference was found between the two models. The direct path was retained to maintain consistent model structure across the two outcomes and to facilitate parallel comparison of mediating mechanisms.

Notably, the chain mediation model tested in this study was constructed based on the IFSMT theoretical framework. Given the cross-sectional study design, the analysis only examines associational patterns among variables and does not imply definitive causal relationships or confirm temporal ordering among variables.

## 3. Results

### 3.1. Common Method Bias Test

The Harman one-factor test was employed to assess common method bias. The analysis revealed the extraction of six common factors with eigenvalues greater than one, and the largest factor accounted for 34.07% of the variance, a figure that fell below the critical threshold of 40% (Podsakoff et al., 2003). This suggests that common method bias was not a significant issue in the present study.

### 3.2. Demographic Characteristics

Overall, 268 pairs of children with autism spectrum disorder and their fathers or mothers were enrolled. The mean age of the children was 5.44 ± 2.60 years, with a range of 3 to 14 years. The mean age of the participating fathers or mothers was 36.83 ± 6.41 years, with a range of 24 to 60 years. Univariate analysis results showed that the total score of difficulties differed significantly by disorder severity, only-child status, residence, education level, employment status, and rehabilitation treatment expenditure. The total score of prosocial behaviour differed significantly with respect to only-child status, residence, education level, employment status, and rehabilitation treatment expenditure. Based on these results, these demographic variables were included as covariates in the subsequent path analysis to adjust for their potential confounding effects. Further details are presented in Table 1.

### 3.3. Descriptive Statistics and Correlation Analysis

The descriptive statistics of mindful parenting, parenting stress, family management, and emotional and behavioural problems are displayed in Table 2. The total difficulties score of children with autism spectrum disorder was 24.72 ± 9.21, with a positive detection rate of 81.3%. Among all dimensions of the total difficulties score, the conduct problems dimension had the highest score of 6.34 ± 3.08, while the emotional symptoms dimension had the lowest score of 6.00 ± 2.91. The positive detection rate was 81.3% for the conduct problems dimension, 76.5% for the emotional symptoms dimension, 79.5% for the peer relationship problems dimension, and the lowest rate of 58.2% for the hyperactivity/inattention dimension. In addition, the mean scores of the total prosocial behaviour score, total mindful parenting score, and total parenting stress score were 5.74 ± 2.61, 60.32 ± 19.35, and 44.61 ± 11.73, respectively, and the positive detection rate of the total prosocial behaviour score was 36.6%.

Correlation analysis results showed that mindful parenting was significantly negatively correlated with parenting stress (*r* = −0.560, *p* < 0.01), negative family management assessment (*r* = −0.412, *p* < 0.01), and total difficulties score (*r* = −0.580, *p* < 0.01). Parenting stress was also significantly positively correlated with negative family management assessment (*r* = 0.402, *p* < 0.01) and total difficulties score (*r* = 0.587, *p* < 0.01). Negative family management assessment was significantly positively correlated with total difficulties score (*r* = 0.511, *p* < 0.01). The results of the correlation analysis are shown in Table 3.

### 3.4. Path Model and Mediating Effect Analysis

Based on the previous literature and the IFSMT framework, the present study specified two path models with observed variables: one with total difficulties score (Model 1) and the other with total prosocial behaviour score (Model 2) as the dependent variable. In both models, mindful parenting served as the independent variable, while parenting stress, positive family management assessment, and negative family management assessment were included as mediating variables. Correlation analysis showed that mindful parenting was significantly correlated with parenting stress, family management variables, total difficulties score, and total prosocial behaviour score, satisfying the prerequisites for mediation analysis. The models were estimated using the MLR method. Both path models demonstrated good fit. The fit indices for Model 1 were as follows: *χ*^2^/*df* = 1.736, RMSEA = 0.052, CFI = 0.963, TLI = 0.952, SRMR = 0.040; the fit indices for Model 2 were: *χ*^2^/*df* = 1.396, RMSEA = 0.038, CFI = 0.978, TLI = 0.973, SRMR = 0.038.

**Figure 2:**
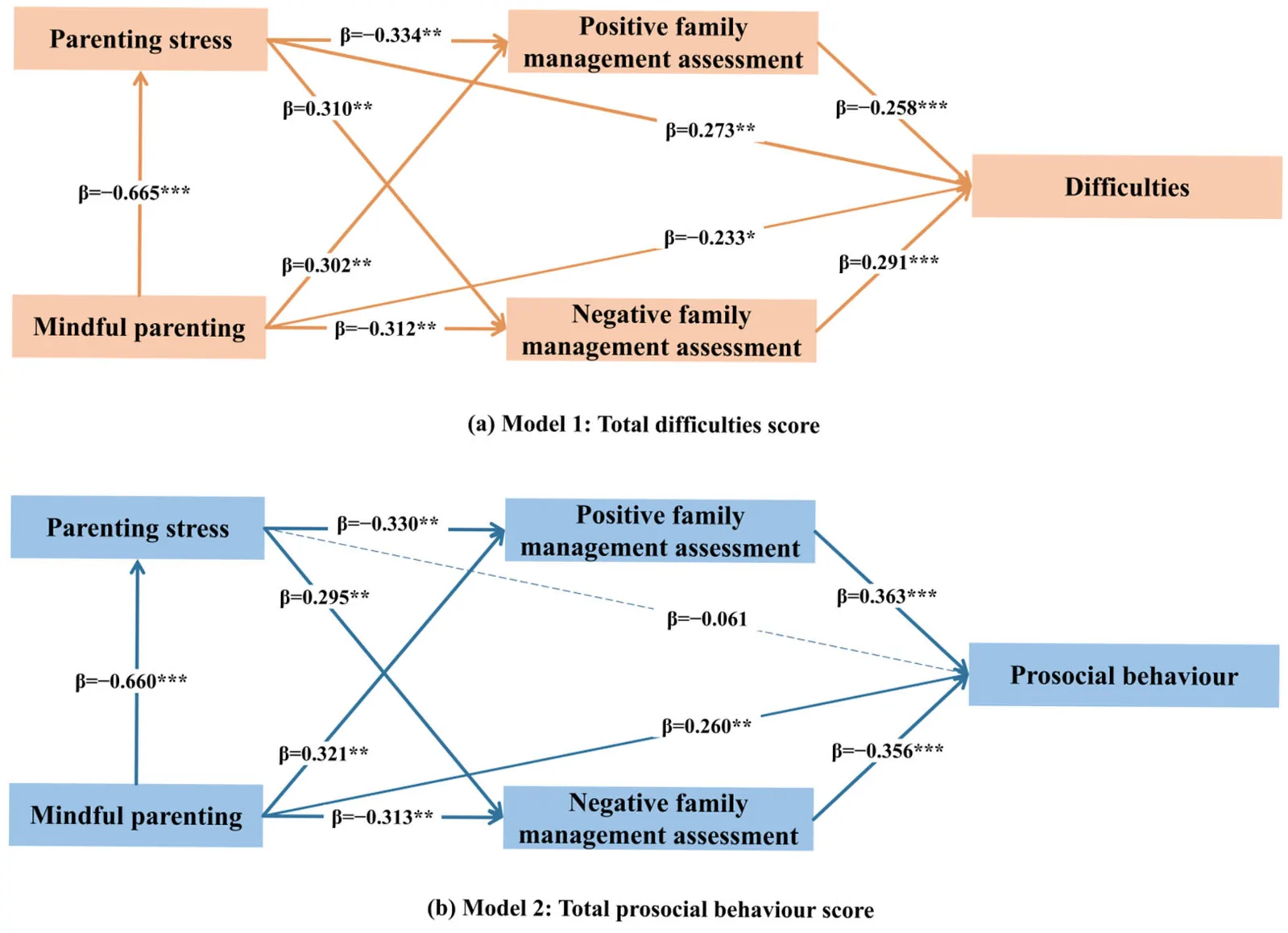
The chain-mediated effects of mindful parenting, parenting stress, and family management on emotional and behavioural problems. Note: * *p* < 0.05, ** *p* < 0.01, *** *p* < 0.001 (two-tailed). Solid lines represent statistically significant paths; dashed lines represent non-significant paths.

As shown in Figure 2, mindful parenting significantly negatively predicted the total difficulties score of children with ASD (*β* = −0.233, *p* < 0.05) and significantly positively predicted their total prosocial behaviour score (*β* = 0.260, *p* < 0.01), supporting Hypothesis 1.

Parenting stress significantly negatively predicted positive family management assessment (Model 1: *β* = −0.334, *p* < 0.01; Model 2: *β* = −0.330, *p* < 0.01) and significantly positively predicted negative family management assessment (Model 1: *β* = 0.310, *p* < 0.01; Model 2: *β* = 0.295, *p* < 0.01). Positive family management assessment significantly negatively predicted total difficulties score (*β* = −0.258, *p* < 0.001) and significantly positively predicted total prosocial behaviour score (*β* = 0.363, *p* < 0.001); negative family management assessment significantly positively predicted total difficulties score (*β* = 0.291, *p* < 0.001) and significantly negatively predicted total prosocial behaviour score (*β* = −0.356, *p* < 0.001). Parenting stress significantly positively predicted total difficulties score (*β* = 0.273, *p* < 0.01).

Regarding the mediation effects, the results for Path 1 (mindful parenting → parenting stress → emotional and behavioural problems in children with ASD) indicated that in Model 1, the indirect effect of this path was significant (*β* = −0.181, *p* = 0.007, 95% *CI*: [−0.313, −0.049]); however, in Model 2, this indirect effect was not significant (*β* = 0.040, *p* = 0.518, 95% *CI*: [−0.081, 0.161]). These findings indicate that parenting stress served as a significant mediator between mindful parenting and difficulties in children with ASD, but the mediating effect on prosocial behaviour was not significant. Therefore, Hypothesis 2 was partially supported.

The examination of Path 2 (mindful parenting → family management → emotional and behavioural problems in children with ASD) showed that in Model 1, the indirect effect of mindful parenting via positive family management assessment was significant (*β* = −0.080, *p* = 0.019, 95% *CI*: [−0.147, −0.013]), and the indirect effect via negative family management assessment was also significant (*β* = −0.091, *p* = 0.022, 95% *CI*: [−0.168, −0.013]); in Model 2, the indirect effect via positive family management assessment was significant (*β* = 0.117, *p* = 0.011, 95% *CI*: [0.026, 0.207]), and the indirect effect via negative family management assessment was likewise significant (*β* = 0.111, *p* = 0.006, 95% *CI*: [0.032, 0.190]). These results confirmed the mediating role of family management between mindful parenting and emotional and behavioural problems in children with ASD, supporting Hypothesis 3.

Path 3 (the chain mediating effect: mindful parenting → parenting stress → family management → emotional and behavioural problems in children with ASD) showed that all chain indirect effects were significant. In Model 1, the chain indirect effect via parenting stress and positive family management assessment was significant (*β* = −0.057, *p* = 0.028, 95% *CI*: [−0.108, −0.006]), and the chain indirect effect via parenting stress and negative family management assessment was also significant (*β* = −0.058, *p* = 0.019, 95% *CI*: [−0.107, −0.009]); in Model 2, the chain indirect effect via parenting stress and positive family management assessment was significant (*β* = 0.079, *p* = 0.016, 95% *CI*: [0.015, 0.144]), and the chain indirect effect via parenting stress and negative family management assessment was likewise significant (*β* = 0.069, *p* = 0.016, 95% *CI*: [0.013, 0.126]). These findings verified the chain mediating roles of parenting stress and family management between mindful parenting and emotional and behavioural problems in children with ASD, supporting Hypothesis 4.

Overall, in both Model 1 (total difficulties score) and Model 2 (prosocial behaviour score), the direct associations between mindful parenting and child outcomes remained statistically significant after accounting for the mediating variables, and the total indirect associations also reached statistical significance. This pattern indicates that parenting stress and family management partially mediate the association between mindful parenting and children’s emotional and behavioural outcomes.

The relevant mediation effect data are presented in detail in Table 4 and Table 5.

## 4. Discussion

This study found that the mindful parenting scores of parents of children with ASD were lower than those reported by Aydin (2023) for parents of young children with ASD aged 3 to 6 years. Three reasons may account for this. First, the age range of children in this study was 3 to 14 years. With increasing age, the stage heterogeneity of ASD symptoms becomes more pronounced, and the frequency and severity of episodes are higher, making it difficult for parents to integrate mindful principles consistently into daily parenting (Northrup et al., 2021). Second, parents of children with ASD in China generally bear a heavy caregiving burden. They not only have to cope with the core symptoms of ASD, comorbid behavioural problems, and the increasing care needs associated with child development, but also endure self-blame, guilt, and shame triggered by social stigma (H. Wang et al., 2022). Third, mindful parenting training and interventions for families affected by ASD have not yet been widely implemented in China, which is also an important reason for the low level of mindful parenting (H. Wang et al., 2022). In conclusion, there is an urgent need to develop mindful parenting interventions and training for parents of children with ASD. Relevant professionals can develop mindful parenting manuals, conduct regular online and offline intervention teaching, and implement longitudinal follow-up to track changes in the mindful parenting of parents dynamically and provide timely professional support.

The results of this study showed that the detection rate of difficulties in children with ASD was 81.3%. Prosocial behaviour refers to voluntary acts intended to benefit others or the group without expectation of external reward; in this study, the abnormal detection rate for prosocial behaviour among children with ASD was 36.6%. However, the abnormal detection rate of emotional and behavioural problems in this study was higher than that reported in previous similar studies in China (Lu et al., 2023). There are two main reasons for this. First, univariate analysis showed that symptom severity was significantly positively correlated with total difficulty scores, with children with moderate-to-severe ASD scoring significantly higher than those with mild ASD. In this study, children with moderate-to-severe ASD accounted for 65.7% of the sample, which is the main reason for the high detection rate. Second, total difficulty scores of children of employed parents were significantly higher than those of children of non-employed parents, suggesting that employed parents who have to balance work and caregiving responsibilities find it difficult to identify and intervene in the emotional and behavioural problems of their children promptly, thus exacerbating the problems. In view of this, it is recommended that multidisciplinary clinical teams collaborate to carry out early identification of emotional and behavioural problems and home-based intervention guidance, and to improve the tiered family support system.

Consistent with previous research (Huang & Wang, 2024), this study found that mindful parenting was significantly negatively correlated with emotional and behavioural problems and significantly positively correlated with prosocial behaviour in children with ASD. Studies by Meppelink et al. (2016) and Hwang et al. (2015) have shown that higher parental mindfulness is associated with better psychopathological symptoms and executive functions in children. The mechanism may be related to the enhancement of central coherence and attentional regulation through mindfulness training. Regarding prosocial behaviour, mindful parenting as a positive parenting style may function through three pathways. First, children imitate the mindful behaviour of their parents, which promotes their capacity for attention allocation and present-moment awareness and increases helping tendencies (Kabat-Zinn, 2023). Second, mindfulness regulates emotions by enhancing prefrontal cortex activity and reducing amygdala reactivity, helping children cope with emotional fluctuations during social interactions (Segal et al., 2012). Third, sustained parent–child mindful interactions create a stable developmental environment, allowing children to gradually internalise prosocial skills (Zelazo & Lyons, 2012). Parents with higher levels of mindful parenting tend to show better perspective-taking, empathic companionship, and emotion regulation modelling, which is associated with better attention control, emotion regulation, and social participation initiative in children. Therefore, mindful parenting should be integrated as a key component of family intervention. Personalised parent–child mindfulness training programmes should be developed based on the severity of the condition of the child, family caregiving resources, the work schedules of parents, and parenting needs, for example through standardised formats such as offline group coaching and online home practice supervision, to continuously reduce emotional and behavioural problems and promote prosocial behaviour.

The study indicated that parenting stress mediated the relationship between mindful parenting and emotional and behavioural problems in children with ASD. A series of studies by Singh on parents of children with developmental disabilities found that mindful parenting training enhanced maternal subjective well-being and interaction satisfaction while reducing aggression, noncompliance, and self-injurious behaviour in children, suggesting that mindful parenting may alleviate parenting stress by increasing parental well-being (Singh et al., 2006, 2007, 2019). Moreover, parenting stress positively predicted problem behaviours in children; parents with higher stress levels tend to create a negative family environment, which in turn adversely affects child behaviour (Baker et al., 2024). Taken together, mindful parenting is associated with lower parenting stress via more positive parent–child interaction and communication and greater caregiver well-being, which corresponds to a healthier family environment and fewer emotional and behavioural problems in children, which aligns with the conclusion of Baker et al. (2024). In contrast, parenting stress failed to mediate the link between mindful parenting and prosocial behaviour despite their significant bivariate correlation (r = −0.492, *p* < 0.01). Simple correlation ignores confounding factors like family management. Once we controlled family management indicators, the independent effect of parenting stress vanished. Parenting stress mainly predicts children’s negative behavioural issues, while prosocial development relies on daily social guidance embedded in family management. Thus, the correlation between parenting stress and prosocial behaviour operates through family management rather than a direct path, which is consistent with previous findings (Singh et al., 2007).

The results showed that family management mediated the relationship between mindful parenting and emotional and behavioural problems in children with ASD. This pathway suggests that mindful parenting is indirectly associated with fewer emotional and behavioural problems via better family management capacity. Family management refers to the process by which family members jointly manage a child’s chronic condition and integrate it into daily life, reflecting the way in which the family copes with and manages ASD symptoms daily. The latest Chinese expert consensus emphasises that the main approach to ASD rehabilitation is intervention that integrates treatment and education in natural everyday settings with family members as the primary agents of implementation (Specialized Committee on Prevention and Treatment of Autism of China Maternal and Child Health Research Association & Hubei Special Children Rehabilitation Association, 2025). This principle highlights the importance of high-quality family care management. Research has shown that the nonjudgmental and present-focused approach advocated by mindful parenting is associated with less excessive parental focus on children’s behavioural differences, lower disorder management burden, and better family management quality, which may ultimately correspond to a lower risk of emotional and behavioural problems, which is consistent with the findings of this study (Hwang et al., 2015; Lu et al., 2023).

Simultaneously, family management mediated the relationship between mindful parenting and prosocial behaviour in children with ASD. Effective family management support is an essential component of lifelong care for individuals with ASD. Mindful parenting can enhance parenting skills and satisfaction with parent–child interaction, motivating parents to actively participate in rehabilitation interventions for their child and to conduct high-quality daily parent–child practice (Singh et al., 2007), thereby improving family management efficacy. In turn, optimised family management helps parents generalise rehabilitation skills to a variety of real-life social scenarios, providing stable support for the social practice of the child and consequently promoting the development of prosocial behaviour (Peng et al., 2025).

Furthermore, this study revealed that parenting stress and family management played a chain mediating role between mindful parenting and emotional and behavioural problems in children with ASD. It is noteworthy that this chain pathway is particularly important in the context of ASD caregiving. Chronically high parenting stress is associated with impaired sleep and cognitive function in caregivers as well as lower caregiving efficiency, which may hinder parents from adopting effective family management strategies (Gnazzo et al., 2026). A study by H. Wang et al. (2020) in China confirmed that parenting stress was negatively correlated with caregiving participation; high stress makes caregivers highly emotionally tense, which in turn impairs their ability to provide optimal care for their children, and caregiving participation is a key component of family management. Thus, if parenting stress is not alleviated, it may directly obstruct the effective operation of family management, thereby exacerbating emotional and behavioural problems in children. Parents who endorse mindful parenting principles of acceptance and present-moment awareness tend to report less emotional exhaustion and lower stress, which is associated with more proactive engagement in symptom management and daily guidance, better family management quality, and better child outcomes. This finding suggests that healthcare professionals should place importance on the dynamic assessment of parenting stress in parents of children with ASD, conduct regular mental health assessments and teach stress reduction techniques, and simultaneously disseminate disorder knowledge and family management skills systematically.

Concurrently, parenting stress and family management also played a chain mediating role between mindful parenting and prosocial behaviour in children with ASD. According to the parenting stress model, negative parent–child interaction exacerbates parenting stress and impairs the social communication ability of the child, whereas parents with high levels of mindful parenting tend to buffer parenting stress through cognitive reappraisal and emotion regulation, which is consistent with previous conclusions (Q. Wang et al., 2023). Family management capacity is also an important prerequisite for intervention compliance and the development of social skills in the child; after receiving targeted intervention, parents can significantly promote the development of prosocial behaviour in the child by improving the quality of parent–child interaction (Minjarez et al., 2025). Taken together, mindful parenting, starting from the alleviation of parenting stress, provides parents with more emotional resources to invest in family management, thereby enhancing family management capacity, and ultimately promoting the improvement of prosocial behaviour in the child through more positive parent–child interaction.

Overall, mindful parenting is associated with fewer emotional/behavioural problems and greater prosocial behaviour in children with ASD. The proposed chain mediation pattern was fully consistent with the data for difficulties, but only partially for prosocial behaviour, where the indirect path via parenting stress was not significant. These differential patterns suggest intervention efforts should target both parenting stress and family management. On the other hand, child health professionals need to improve the family-centred disorder management support system by offering standardised health education and skills training to enhance the parent–child interaction skills of caregivers and the level of family empowerment, while also tailoring personalised intervention plans to the roles of mothers and fathers, guiding parents to effectively mediate social interventions for their children under professional supervision, to continuously promote the development of prosocial behaviour.

## 5. Limitations

There were several limitations in this study. First, the study relied on self-reported data, which may introduce social desirability and recall biases. Second, due to the cross-sectional design, causal relationships and temporal ordering among variables cannot be definitively established. The chain mediation model tested in this study is based on theoretical assumptions and only reflects associational patterns among variables, rather than confirmed causal pathways. Furthermore, bidirectional relationships may exist between study variables. For example, children’s emotional and behavioural problems may in turn elevate parenting stress and impair the quality of family management, forming a reciprocal dynamic (O’Laughlin et al., 2018). The current study only examined the theoretically proposed unidirectional pathway and cannot disentangle bidirectional effects. Longitudinal cross-lagged designs are recommended in future research to clarify the direction of effects. Third, this study examined the influencing mechanisms solely from the family perspective, without considering other potentially significant factors at the school or peer level. Future research could include multi-level variables to provide a more comprehensive and nuanced understanding of the complex interplay underlying these outcomes. Finally, the convenience sampling method, which recruited participants only from tertiary hospitals and special education institutions in Jinan, limits the representativeness of the sample. The findings may not be generalisable to all families of children with ASD, such as those in other geographic regions, those not receiving formal rehabilitation services, or those in different socioeconomic contexts. Future studies adopting multi-centre stratified sampling across diverse regions are recommended to improve sample representativeness and enhance the generalisability of the results.

## 6. Conclusions

The present study indicates that mindful parenting is associated with emotional and behavioural problems and prosocial behaviour in children with ASD, and that parenting stress and family management mediate and chain-mediate these associations. The findings reveal that parents of children with ASD exhibit relatively low levels of mindful parenting, while the emotional and behavioural problems of the children remain severe, highlighting a situation that warrants serious attention. This study offers an understanding of the mechanisms underlying the relationship between mindful parenting and children’s emotional and behavioural outcomes, and provides a certain theoretical and practical direction for future efforts aimed at reducing children’s emotional and behavioural problems, enhancing mindful parenting, and promoting the collaborative improvement of both parental and child health outcomes. Clinical practitioners and rehabilitation institutions should establish targeted, family-centred mindful parenting intervention programmes, whilst simultaneously developing robust measures that address both the reduction of parenting stress and the enhancement of family management capacity, thereby achieving meaningful improvements in the emotional and behavioural outcomes of children with ASD.

## 7. Implications for Practice and Future Research

The findings carry meaningful implications for both clinical rehabilitation practice and future research. Mindful parenting, parenting stress and family management are modifiable factors within daily family care and interaction; multidisciplinary teams should thus incorporate routine assessment of parental mindful parenting and parenting stress into standard care pathways for early identification of high-need families, deliver tiered support from brief mindfulness practices for acute caregiving distress to structured group programmes for sustained skill building, guide parents to integrate condition management into naturalistic daily routines and develop responsive interaction skills to foster children’s emotional and behavioural regulation, and establish clinic-to-home transitional care with dynamic monitoring to adjust family support plans promptly. For future research, given the cross-sectional design and reliance on self-report in this study, longitudinal or interventional designs are needed to clarify causal and temporal associations; further work may also complement self-report data with objective behavioural observation and physiological or neural correlates of dyadic parent–child interaction, adopt a bidirectional perspective to examine mutual parent–child shaping processes, and include school-, peer- and community-level factors to build a more nuanced multi-level understanding of emotional and behavioural development in children with ASD.

## Figures and Tables

**Table 1:** Demographic characteristics and univariate analysis of difficulties and prosocial behaviour total scores (N = 268).

Variable	Category	Number (%)	Difficulties			Prosocial Behaviour		
			Score	*t*/*F*	*p*	Score	*t*/*F*	*p*
**Children with autism spectrum disorder**								
Gender	Male	194 (72.4)	24.29 ± 9.43	1.24 ^a^	0.215	5.81 ± 2.61	−0.72 ^a^	0.475
	Female	74 (27.6)	25.85 ± 8.56			5.55 ± 2.61		
Disorder duration (years)	<3	135 (50.4)	24.34 ± 9.31	−0.68 ^a^	0.498	5.97 ± 2.55	1.47 ^a^	0.143
	≥3	133 (49.6)	25.11 ± 9.12			5.50 ± 2.66		
Severity of disorder	Mild	92 (34.3)	19.61 ± 8.79	−7.02 ^a^	<0.001	6.07 ± 2.32	1.48 ^a^	0.139
	Moderate/Severe	176 (65.7)	27.39 ± 8.27			5.57 ± 2.74		
Only child or not	Yes	132 (49.3)	23.05 ± 9.75	2.96 ^a^	0.003	6.32 ± 2.54	−3.07 ^a^	0.002
	No	136 (50.7)	26.34 ± 8.38			5.26 ± 2.60		
**Parents of children with autism spectrum disorder**								
Gender	Male	60 (22.4)	23.38 ± 9.01	1.28 ^a^	0.202	5.58 ± 2.70	0.52 ^a^	0.601
	Female	208 (77.6)	25.11 ± 9.25			5.78 ± 2.59		
Residence	Urban	145 (54.1)	22.26 ± 9.59	−5.03 ^a^	<0.001	6.62 ± 2.36	6.45 ^a^	<0.001
	Rural	123 (45.9)	27.62 ± 7.84			4.70 ± 2.51		
Education level	Junior high school and below	58 (21.6)	25.95 ± 9.29	5.20 ^b^	0.006	4.93 ± 2.53	27.04 ^b^	<0.001
	Senior high school/Technical secondary school	82 (30.6)	26.73 ± 9.01			4.57 ± 2.64		
	Junior college/Bachelor’s degree and above	128 (47.8)	22.88 ± 9.00			6.85 ± 2.13		
Employment status	Employed (full-time, part-time)	163 (60.8)	27.04 ± 8.66	5.39 ^a^	<0.001	5.26 ± 2.73	−4.03 ^a^	<0.001
	Retired/Resigned/Unemployed	105 (39.2)	21.12 ± 8.91			6.49 ± 2.23		
Average monthly income (CNY/USD)	<2000 CNY (<278 USD)	61 (22.8)	26.30 ± 9.19	1.81 ^b^	0.146	5.39 ± 2.56	1.96 ^b^	0.120
	2000–4999 CNY (278–694 USD)	89 (33.2)	23.34 ± 9.34			6.27 ± 2.33		
	5000–7999 CNY (694–1111 USD)	79 (29.5)	24.22 ± 9.44			5.59 ± 2.63		
	≥8000 CNY (≥1111 USD)	39 (14.6)	26.44 ± 8.12			5.36 ± 3.08		
Rehabilitation treatment expenditure (CNY/USD)	<2000 CNY (<278 USD)	100 (37.3)	28.84 ± 6.55	18.77 ^b^	<0.001	5.17 ± 2.76	5.21 ^b^	0.006
	2000–4000 CNY (278–556 USD)	87 (32.5)	23.02 ± 9.11			5.77 ± 2.36		
	>4000 CNY (>556 USD)	81 (30.2)	21.46 ± 10.28			6.41 ± 2.53		
Duration of continuous care (years)	<3	97 (36.2)	24.33 ± 9.59	−0.52 ^a^	0.602	5.95 ± 2.59	0.99 ^a^	0.322
	≥3	171 (63.8)	24.94 ± 9.01			5.62 ± 2.62		

^a^ t-value; ^b^ F-value.

**Table 2:** Descriptive statistics of mindful parenting, parenting stress, family management, and emotional and behavioural problems (N = 268).

Variable	Mean	Standard Deviation	Minimum	Maximum	Number of Positive Cases	Detection Rate
**Total difficulties score**	24.72	9.21	1	40	218	81.3
Emotional symptoms	6.00	2.91	0	10	205	76.5
Conduct problems	6.34	3.08	0	10	218	81.3
Hyperactivity/Inattention	6.31	3.02	0	10	156	58.2
Peer relationship problems	6.07	2.77	0	10	213	79.5
**Total prosocial behaviour score**	5.74	2.61	1	10	98	36.6
**Total mindful parenting score**	60.32	19.35	29	107	/	/
Being present with mindfulness	27.94	9.66	13	50	/	/
Mindful discipline	32.38	10.98	15	57	/	/
**Total parenting stress score**	44.61	11.73	19	71	/	/
Parental distress	13.51	4.94	5	25	/	/
Parent–child dysfunctional interaction	15.13	4.62	6	24	/	/
Difficult child	15.97	4.35	6	25	/	/
**Total positive family management assessment score**	77.54	21.36	34	117	/	/
Child daily life	16.19	6.64	5	25	/	/
Condition management ability	36.43	9.32	16	58	/	/
Parental mutual support	24.92	8.72	8	40	/	/
**Total negative family management assessment score**	86.27	20.39	34	124	/	/
Condition-related worry	30.89	7.92	12	49	/	/
Condition management difficulty	42.50	11.45	17	66	/	/
Condition management burden	12.88	4.29	5	19	/	/

**Table 3:** The correlation between mindful parenting, parenting stress, family management, and emotional and behavioural problems (N = 268).

Variables	Mindful Parenting	Parenting Stress	Positive Family Management Assessment	Negative Family Management Assessment	Total Difficulties Score	Total Prosocial Behaviour Score
Mindful parenting	1					
Parenting stress	−0.560 **	1				
Positive family management assessment	0.463 **	−0.447 **	1			
Negative family management assessment	−0.412 **	0.402 **	−0.315 **	1		
Total difficulties score	−0.580 **	0.587 **	−0.517 **	0.511 **	1	
Total prosocial behaviour score	0.559 **	−0.492 **	0.553 **	−0.522 **	−0.447 **	1

** *p* < 0.01 (two-tailed).

**Table 4:** Standardized path coefficients of the model.

Independent Variable	Dependent Variable	*β*	*S.E.*	95% *CI*	*p*
**Model 1: Total difficulties score**					
Mindful parenting	Difficulties	−0.233	0.100	−0.428, −0.037	0.020
Parenting stress	Difficulties	0.273	0.103	0.072, 0.474	0.008
Positive family management assessment	Difficulties	−0.258	0.068	−0.391, −0.125	<0.001
Negative family management assessment	Difficulties	0.291	0.077	0.140, 0.442	<0.001
Mindful parenting	Positive family management assessment	0.310	0.105	0.104, 0.517	0.003
Parenting stress	Positive family management assessment	−0.334	0.105	−0.541, −0.127	0.002
Mindful parenting	Negative family management assessment	−0.312	0.099	−0.506, −0.118	0.002
Parenting stress	Negative family management assessment	0.302	0.102	0.101, 0.503	0.003
Mindful parenting	Parenting stress	−0.665	0.054	−0.770, −0.560	<0.001
**Model 2: Total prosocial behaviour score**					
Mindful parenting	Prosocial behaviour	0.260	0.087	0.089, 0.432	0.003
Parenting stress	Prosocial behaviour	−0.061	0.095	−0.246, 0.125	0.522
Positive family management assessment	Prosocial behaviour	0.363	0.079	0.209, 0.518	<0.001
Negative family management assessment	Prosocial behaviour	−0.356	0.067	−0.487, −0.225	<0.001
Mindful parenting	Positive family management assessment	0.321	0.104	0.117, 0.526	0.002
Parenting stress	Positive family management assessment	−0.330	0.104	−0.535, −0.126	0.002
Mindful parenting	Negative family management assessment	−0.313	0.098	−0.505, −0.120	0.001
Parenting stress	Negative family management assessment	0.295	0.102	0.096, 0.495	0.004
Mindful parenting	Parenting stress	−0.660	0.055	−0.767, −0.553	<0.001

**Table 5:** Decomposition of mediating effects of the model.

Model Path	Effect	95% *CI*	*p*
**Model 1: Total difficulties score**			
Total effects	−0.701	−0.799, −0.602	<0.001
Total indirect effect	−0.468	−0.607, −0.329	<0.001
Mindful parenting → Parenting stress → Total difficulties score	−0.181	−0.313, −0.049	0.007
Mindful parenting → Positive family management assessment → Total difficulties score	−0.080	−0.147, −0.013	0.019
Mindful parenting → Negative family management assessment → Total difficulties score	−0.091	−0.168, −0.013	0.022
Mindful parenting → Parenting stress → Positive family management assessment → Total difficulties score	−0.057	−0.108, −0.006	0.028
Mindful parenting → Parenting stress → Negative family management assessment → Total difficulties score	−0.058	−0.107, −0.009	0.019
Direct effects	−0.233	−0.428, −0.037	0.020
**Model 2: Total prosocial behaviour score**			
Total effects	0.677	0.586, 0.767	<0.001
Total indirect effect	0.416	0.279, 0.553	<0.001
Mindful parenting → Parenting stress → Total prosocial behaviour score	0.040	−0.081, 0.161	0.518
Mindful parenting → Positive family management assessment → Total prosocial behaviour score	0.117	0.026, 0.207	0.011
Mindful parenting → Negative family management assessment → Total prosocial behaviour score	0.111	0.032, 0.190	0.006
Mindful parenting → Parenting stress → Positive family management assessment → Total prosocial behaviour score	0.079	0.015, 0.144	0.016
Mindful parenting → Parenting stress → Negative family management assessment → Total prosocial behaviour score	0.069	0.013, 0.126	0.016
Direct effects	0.260	0.089, 0.432	0.003

## Data Availability

The data presented in this study are available on request from the corresponding author due to ethical restrictions (the need to protect participant confidentiality and comply with institutional review board policies).

## Abbreviations

The following abbreviations are used in this manuscript:

ASD	Autism Spectrum Disorder
CFA	Confirmatory Factor Analysis
CFI	Comparative Fit Index
DSM-5	Diagnostic and Statistical Manual of Mental Disorders, 5th edn.
FaMM	Family Management Measure
IFSMT	Individual and Family Self-Management Theory
MIPQ	Mindfulness in Parenting Questionnaire
MLR	Robust Maximum Likelihood
PSI-SF-15	Parenting Stress Index-Short Form (15 items)
RMSEA	Root Mean Square Error of Approximation
SDQ	Strengths and Difficulties Questionnaire
SRMR	Standardized Root Mean Square Residual
STROBE	Strengthening the Reporting of Observational Studies in Epidemiology
TLI	Tucker–Lewis Index
*x*^2^/*df*	Chi-Square to Degrees of Freedom Ratio
